# Supplementary material for: Natural Products Induce Different Anti-Tumor Immune Responses in Murine Models of 4T1 Mammary Carcinoma and B16-F10 Melanoma
Source: Int J Mol Sci. 2023 Nov 24;24(23):16698. doi: 10.3390/ijms242316698 (PMC10706186; doi:10.3390/ijms242316698)
Supplement: Supplementary file 1 [file ijms-24-16698-s001.zip › ijms-2695171-supplementary.pdf]

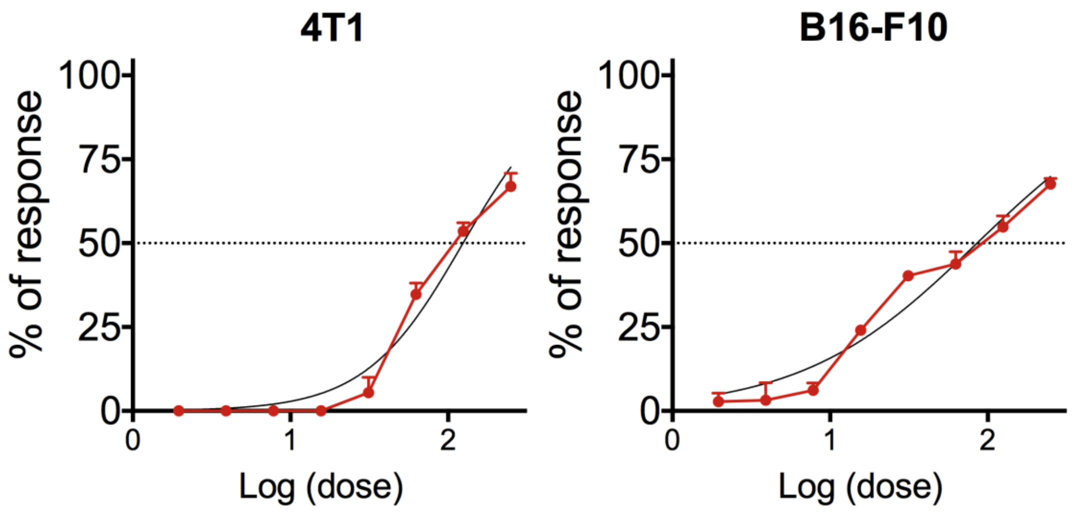

Figure S1. Dose–response viability curve. Cells were seeded in 96-well plates and treated with different concentrations of *P. nigrum* extract for 48 h. Viability were determined by the MTT method described in Materials and Methods. The IC_{50} value was calculated using GraphPad Prism version 8.1.1 for Mac OS X statistics software (GraphPad Software, San Diego, CA). Black line: nonlinear regression curve fitting.

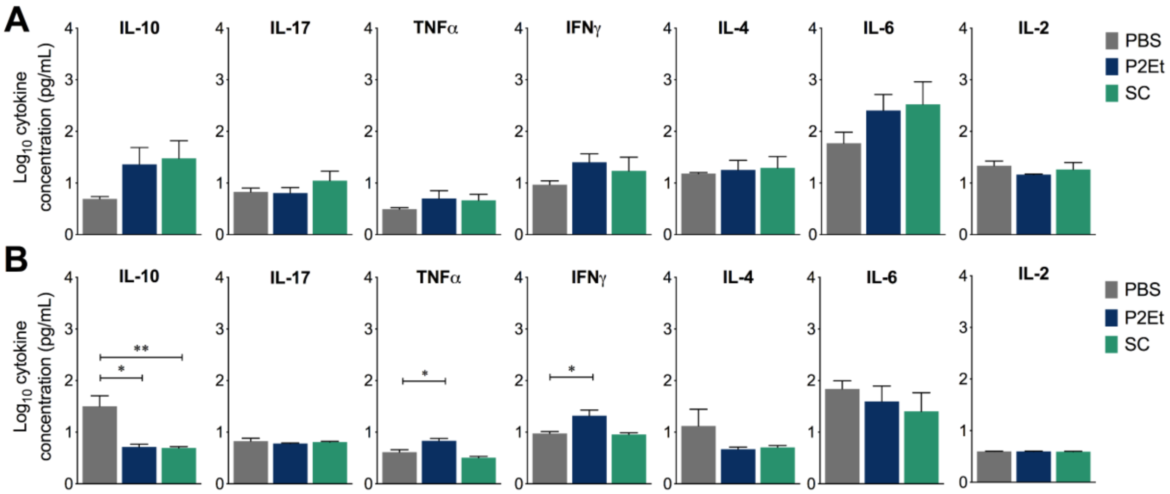

Figure S2. Cytokine levels in the serum of 4T1 tumor-bearing mice **(A)** or B16-F10 tumor-bearing mice **(B)** treated with Anamu-SC, P2Et, or PBS (control). The data were log-transformed and plotted as the mean ± SEM.

Table S1. List of antibodies for flow cytometry

Target	Conjugate	Clone	Company	Multicolor panel
CD45	PE-Cy5	30-F11	Biolegend	1
CD3	Pacific Blue	17A2	Biolegend	1
CD4	Brilliant Violet 570	RM4-5	Biolegend	1
CD8	PE-Dazzle 594	53.6.7	Biolegend	1
CD11c	FITC	HL3	BD Biosciencies	1
CD11b	Alexa Fluor 700	M1/70	Biolegend	1
Ly6C	APC-Cy7	AL-21	Biolegend	1
Ly6G	PE-Cy7	1A8	Biolegend	1
CD45	PE-Cy5	30-F11	Biolegend	2
CD3	Pacific Blue	17A2	Biolegend	2
CD4	Brilliant Violet 570	RM4-5	Biolegend	2
CD8	PE-Dazzle 594	53.6.7	Biolegend	2
CD44	PE-Cy7	IM7	Biolegend	2
CD25	APC	3C7	Biolegend	2
CTLA-4	PE	UC10-4F10-11	Biolegend	2
FoxP3	FICT	MF23	BD Biosciencies	2
CD45	PE-Cy5	30-F11	Biolegend	3
CD3	Pacific Blue	17A2	Biolegend	3
CD4	Brilliant Violet 570	RM4-5	Biolegend	3
CD8	PE-Dazzle 594	53.6.7	Biolegend	3
IFNγ	Alexa Fluor 700	XMG1.2	BD Biosciencies	3
TNFα	PE-Cy7	MP6-XT22	BD Biosciencies	3
IL-2	FITC	JES6-5H4	BD Biosciencies	3
Perforin	APC	S16009A	Biolegend	3
Granzyme B	PE	QA16A02	Biolegend	3
